# Food Neophobia in Celiac Disease and Other Gluten-Free Diet Individuals

**DOI:** 10.3390/nu11081762

**Published:** 2019-07-31

**Authors:** Wioleta Zysk, Dominika Głąbska, Dominika Guzek

**Affiliations:** 1 Department of Organization and Consumption Economics, Faculty of Human Nutrition and Consumer Sciences, Warsaw University of Life Sciences (SGGW–WULS), 159C Nowoursynowska Street, 02-787 Warsaw, Poland; 2 Department of Dietetics, Faculty of Human Nutrition and Consumer Sciences, Warsaw University of Life Sciences (SGGW–WULS), 159C Nowoursynowska Street, 02-787 Warsaw, Poland

**Keywords:** Food Neophobia Scale (FNS), celiac disease, gluten-free diet, adults, eating behaviors, consumer behaviors

## Abstract

The only treatment currently available to combat celiac disease (CD) is strict adherence to a gluten-free diet (GFD), but there may be various determinants of its adherence, including food neophobia (FN), that is associated with sensory aversions, or fears of negative consequences of eating specific food products, that may be crucial for CD patients following a GFD. The aim of the present study was to analyze food neophobia levels and its determinants in CD patients in comparison with other individuals who follow a GFD based on their own decision. The study was conducted in two independent groups of individuals following a GFD: those diagnosed with CD (*n* = 101) and those following a GFD based on their own decision (*n* = 124). Each group was recruited with cooperation from the local CD and GFD societies located in Poland. The FN was assessed using the Food Neophobia Scale (FNS) and compared between groups, as well as the influence of gender, age, body mass index, educational level, place of residence and employment status was assessed. It was stated, that for the individuals following a GFD, CD was the major determinant of FN. The FNS score values were higher (indicating higher food neophobia) for CD individuals (39.4 ± 9.2), than for those following a GFD based on their own decision (33.6 ± 8.7; *p* < 0.0001) and it was observed both for general group and for sub-groups stratified by assessed variables. Moreover, the indicated variables did not influence the FNS in any of the analyzed groups. The influence of CD with no influence of other variables was confirmed in the regression analysis. It may be concluded that CD is a major contributor to FN, which can be attributed to fear of developing adverse reactions to gluten-contaminated food products, which is more pronounced in CD patients compared to non-CD patients following a GFD based on their own decision.

## 1. Introduction

Food neophobia is described as a reluctance to try novel or unknown food products [1], and individuals with high food neophobia levels may be indicated as a specific group of consumers [2], which can be mainly attributed to their sensory aversions, as well as to the fear of negative consequences that can occur following the consumption of specific food products [3]. The latter reason may be especially important for patients diagnosed with diet-related diseases, particularly for those who develop food allergies, intolerances, and other adverse reactions to food products, including celiac disease (CD).

CD is a chronic small intestinal immune-mediated enteropathy precipitated by exposure to dietary gluten in genetically predisposed individuals [4]. It is characterized by an aberrant adaptive immune response to gluten, a protein found in wheat, barley, rye [5], and probably to oats [6]. Gluten consumption by CD patients causes intestinal enteropathy which is associated with an impairment of the mucosal surface, resulting in gastrointestinal symptoms and abnormal absorption of nutrients [7]. The only treatment currently available to combat CD is strict adherence to a gluten-free diet (GFD), which subsequently relieves symptoms [8], allows mucosal healing [9], and reduces the risk of complications, such as osteopenia and osteoporosis [10], anemia [8], as well as mood disorders [11].

However, following a GFD is associated with increased restrictions, making it burdensome and challenging to adhere, and is also known to be associated with some economic burden [12], due to the fact that gluten-free products (GFPs) are expensive in many countries and only a narrow range of products are available, which are often difficult to obtain. Only a few countries (e.g., Canada, Italy, and the United Kingdom) have a policy to reduce the financial burden of CD patients—by providing tax reductions for the extra cost of GFPs, vouchers for CD patients for buying GFPs, or GFP prescriptions for CD patients [13]—which may influence the quality of life of CD patients. Additionally, there are also other factors that are known to restrict the adherence to a GFD, which include the necessity to rely on the information provided in restaurants and labels of the packaged food products (including their certification as a GFP), as well as psychological barriers to GFD adherence and diet reliance [14].

CD patients should also avoid food products contaminated with even small amounts of gluten [15]. Even if the product is naturally gluten-free, there is always a risk of contamination with gluten-containing cereals, especially in case of non-certified products and dishes at restaurants [16]. Therefore, the selection of food products for CD patients is quite challenging, resulting from their fear and avoidance of unsafe food products. Considering that a number of new food products appear on the market each year [17], CD patients must always make a fact-based decision about consuming them; however, there are also other factors that interfere with the decisions that determine food consumption, including food neophobia. This is associated with the fact that food neophobic and food neophilic individuals seem to perceive unknown food products in a different way [18]. Neophobic individual’s food product choices are more complex and elaborate; they may incorporate more personal values into the evaluation of a new product [19], which for CD patients may be crucial for the process of choosing food products.

The determinants of food neophobia in CD patients and patients with other diet-related diseases are still unknown. There is a possibility that the diet-related disease is one such determinant, as it is hypothesized that food neophobia may be one of the mechanisms that protects an organism from consuming potentially unsafe or dangerous food products [20]. Especially for some CD patients, disordered eating behaviors are observed [21] that may be attributed to food neophobia and may be stated as typical for them [22]. 

It must be mentioned that individuals with food neophobia may reduce the consumption of some health-promoting products [23], and as a result, it may be associated with an increased risk of obesity and non-communicable diseases [24]. Taking this into account, it must be emphasized that knowledge about food neophobia in patients following an elimination diet would allow to properly formulate the dietary recommendations for them. 

Till date, no study has analyzed the determinants of food neophobia in CD patients; however, two studies by Satherley [22,25] included the Food Neophobia Scale (FNS) to measure the prevalence and predictors of disordered eating behavior in women with CD [22], as well as to develop the Celiac Disease Food Attitudes and Behaviors Scale (CD-FAB) [25]. Therefore, the aim of the present study was to analyze food neophobia levels and its determinants in CD patients in comparison with other individuals who follow a GFD based on their own decision.

## 2. Materials and Methods 

### 2.1. Ethics Approval Statement

The study was conducted according to the guidelines laid down in the Declaration of Helsinki. The study was approved by the Ethics Committee of the Faculty of Human Nutrition and Consumer Sciences of the Warsaw University of Life Sciences (No. 20/2017; 19.06.2017). All the participants provided their informed consent to participate.

### 2.2. Study Participants and Analyzed Variables

The study was conducted in two independent groups of individuals following a GFD: those diagnosed with CD and those following a GFD based on their own decision. Each group was recruited with cooperation from the local CD and GFD societies located in Poland. Moreover, inclusion and exclusion criteria for recruitment were specific for each group.

For the individuals following a GFD and diagnosed with CD (CD-GFD group), inclusion criteria were as follows: Polish Caucasian individuals, aged 18–80 years, diagnosed with CD and biopsy-confirmed by a physician [26], following a GFD for at least 6 months, declared being responsible for meal preparation at home, and declared being responsible for purchasing of GFPs.

For CD-GFD group, exclusion criteria were as follows: not providing informed consent to participate, and any missing data in the FNS questionnaire. 

For the individuals following GFD and with no CD diagnosed, but following GFD based on their own decision (non-CD-GFD group), inclusion criteria were as follows: Polish Caucasian individuals, aged 18–80 years, diagnosis of CD excluded by a physician, diagnosis of other gluten-related diseases (non-celiac gluten sensitivity, gluten ataxia, wheat allergy, and dermatitis herpetiformis) excluded by a physician, following GFD for at least 6 months, based on their own decision, declared being responsible for meal preparation at home, declared being responsible for purchasing GFPs.

For non-CD-GFD group, exclusion criteria were as follows: not providing informed consent to participate, and any missing data in the FNS questionnaire.

**Figure 1:**
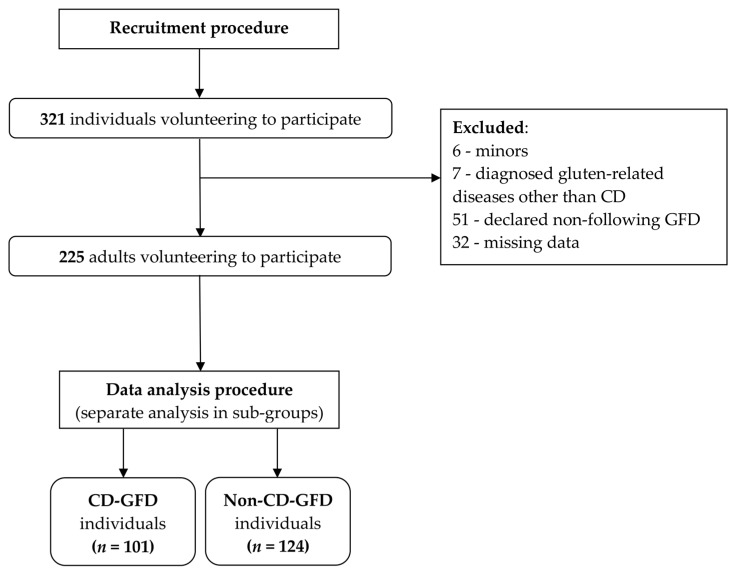
Participants included to the study.

After the screening process, 225 individuals were included in the study, and divided into two sub-groups based on the confirmed diagnosis of CD (Figure 1).

In order to assess the possible variables influencing the food neophobia level, the following characteristics were included:

- gender (stratified into sub-groups of male and female individuals),
- age (analyzed as a continuous variable),
- body mass index (BMI), calculated based on weight (kg) and height (m^2^) [27] ((1) stratified into sub-groups as: below 18.5 underweight; 18.5–24.9 normal; above 25 excessive body mass; (2) analyzed as a continuous variable),
- educational level (stratified into sub-groups as primary and secondary education, tertiary education, and university degree holders),
- place of residence (stratified into sub-groups as villages, towns of <20,000 residents, cities of 20,000–100,000 residents, and cities of >100,000 residents), and
- employment status (stratified into sub-groups of employed and non-employed individuals, including unemployed, students, and retired).

The computer-assisted web interview (CAWI) method was applied and the questionnaires were distributed in cooperation with CD associations and GFD associations.

### 2.3. Assessment of Food Neophobia Level

**Figure 2:**
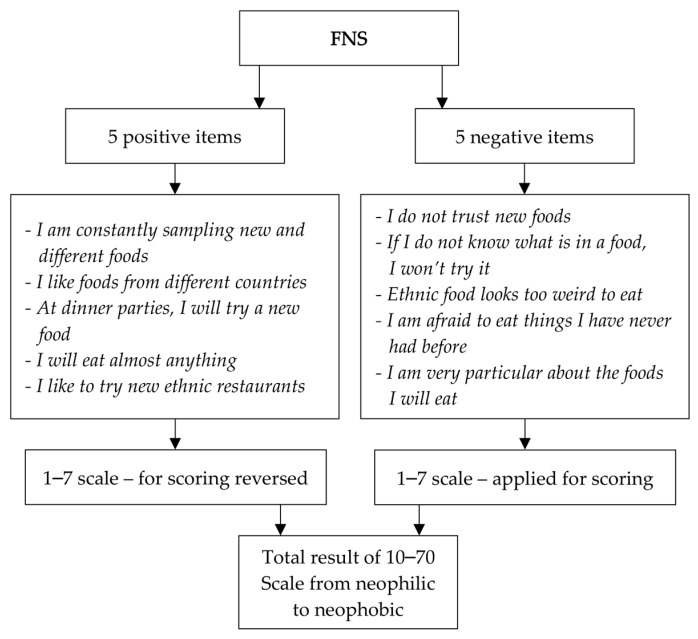
Food Neophobia Scale applied in the conducted study.

The food neophobia level was analyzed using the FNS developed by Pliner and Hobden [28], which is a 10-item scale (Figure 2) that has been applied worldwide to predict willingness to try new foods [29,30,31]. In the present study, the FNS exhibited a respectable level of internal consistency when measured by Cronbach’s alpha [32], both for CD-GFD individuals (0.74) and non-CD-GFD individuals (0.79). 

This scale demonstrated good reliability and validity for the measurement of food neophobia level among the participants [33]. The Polish translation [34], being after transcultural adaptation, of the original scale was positively validated in a Polish population with good internal consistency [23] and was applied nationwide for research purposes among Polish residents [35,36], but was not applied for CD patients. 

In order to analyze the food neophobia level among the participants in the present study, the FNS score was assessed using two different methods:

- as a continuous variable for the obtained score (number of points), ranging from 10 to 70, as is commonly applied for the precise assessment of the FNS score [37,38], and
- as relative FNS categories within sub-groups, after stratifying the participants in both sub-groups into three categories, representing low food neophobia level (for the FNS score lower than the mean value – standard deviation (SD)), average food neophobia level (for the FNS score within the range from mean value – SD to mean value + SD), and high food neophobia level (for the FNS score higher than mean value + SD), which is commonly applied for analysis [36,39,40].

### 2.4. Statistical Analysis

The statistical analysis included:

- comparison of food neophobia levels between the sub-groups of GFD patients, between CD and non-CD patients (CD-GFD vs. non-CD-GFD), and
- assessment of the possible variables influencing the food neophobia levels in CD-GFD and non-CD-GFD sub-groups, as well as in the total group of GFD respondents (with the following variables included: gender, age, BMI, educational level, place of residence, and employment status).

The sample size was calculated for the confidence level of 95% and margin of error of 10%. To assess the internal consistency of the FNS, Cronbach’s alpha coefficient was applied. 

The distributions of the analyzed data were verified using the Shapiro–Wilk test to detect whether a parametric distribution was observed. For parametric distributions, differences between the groups were identified using Student’s *t*-test (for two groups) or one-way analysis of variance (for more than two groups). For non-parametric distributions, differences between the groups were identified using the Mann–Whitney U test (for two groups) or the Kruskal–Wallis test (for more than two groups). Analysis of correlation was performed using Pearson’s correlation (for parametric distributions) and Spearman’s rank correlation (for non-parametric distributions). Differences between the categorical data were identified using the Chi-squared test. 

Afterwards, additional analysis of multiple regression in a backward stepwise model was conducted for the following variables: CD, gender, age, BMI, educational level, place of residence, and employment status as determinants of FNS score.

The significance threshold was set at 0.05. Statistical analysis was conducted using the following software: Statistica 13.0 (StatSoft Inc., Tulsa, OK, USA) and Statgraphics Plus for Windows 4.0 (Statgraphics Technologies Inc., The Plains, VA, USA).

## 3. Results

### 3.1. Characteristics of the Study Sample

Characteristics of the study subjects are presented in Table 1. There was no difference between two sub-groups (CD-GFD and non-CD-GFD), except for the age, as the median value for the CD-GFD group was found to be lower by two years, than for the non-CD-GFD group.

### 3.2. Comparison of Food Neophobia between CD and Non-CD Individuals Following a GFD

The FNS scores of the respondents in sub-groups of CD-GFD and non-CD-GFD individuals are presented in Table 2. The observed scores showed a significant difference between the sub-groups, where CD-GFD individuals showed higher values (higher food neophobia) when compared to non-CD-GFD subjects. Moreover, share of respondents in relative food neophobia categories within sub-groups for CD-GFD and non-CD-GFD individuals did not differ. The proportion of individuals showing high FNS scores, defined as 1.0 SD above the mean value for the studied sub-sample, was similar between the sub-groups (approximately 15% of the sub-group). 

### 3.3. Determinants of Food Neophobia in CD and Non-CD Individuals Following a GFD 

The FNS scores stratified by gender in the sub-groups of CD-GFD and non-CD-GFD individuals are presented in Table 3. The observed scores differed significantly between CD-GFD and non-CD-GFD individuals, both for female and male individuals, but no gender-related differences were noted in the subjects within the CD-GFD and non-CD-GFD groups. Both male and female participants of the CD-GFD group demonstrated higher FNS scores (higher food neophobia) compared to their non-CD-GFD counterparts.

The results of correlation analysis between FNS scores and age, as well as BMI, in the sub-groups of CD-GFD and non-CD-GFD individuals are presented in Table 4. No significant correlation was observed between the FNS scores and both age and BMI parameters, both for CD-GFD and non-CD-GFD individuals.

The FNS scores stratified by BMI in the sub-groups of CD-GFD and non-CD-GFD individuals are presented in Table 5. The observed scores differed significantly between CD-GFD and non-CD-GFD individuals for normal-weight and excessive body mass participants, but not for the underweight BMI sub-group. Moreover, no BMI-related differences were noted in the subjects within the CD-GFD and non-CD-GFD groups. Normal-weight and excessive body mass participants of the CD-GFD group demonstrated higher FNS scores (higher food neophobia) compared to their non-CD-GFD counterparts.

The FNS scores stratified by educational level in the sub-groups of CD-GFD and non-CD-GFD individuals are presented in Table 6. The observed scores differed significantly between CD-GFD and non-CD-GFD individuals only for participants with a university degree, but not for other sub-groups of educational level. Moreover, no education-related differences were noted in the subjects within the CD-GFD and non-CD-GFD groups. Participants with a university degree in the CD-GFD group demonstrated higher FNS scores (higher food neophobia) compared to their non-CD-GFD counterparts.

The FNS scores stratified by place of residence in the sub-groups of CD-GFD and non-CD-GFD individuals are presented in Table 7. The observed scores differed significantly between CD-GFD and non-CD-GFD individuals only for participants from big cities, but not for other place of residence subgroups. Moreover, no residence-related differences were noted in the subjects within the CD-GFD and non-CD-GFD groups. Participants from big cities in the CD-GFD group demonstrated higher FNS scores (higher food neophobia) compared to their non-CD-GFD counterparts.

The FNS scores stratified by employment status in the sub-groups of CD-GFD and non-CD-GFD individuals are presented in Table 8. The observed scores differed significantly between CD-GFD and non-CD-GFD individuals only for employed participants, but not for other sub-groups of employment status. Moreover, no employment-related differences were noted in the subjects within the CD-GFD and non-CD-GFD groups. Employed participants of the CD-GFD group demonstrated higher FNS scores (higher food neophobia) compared to their non-CD-GFD counterparts.

The additional multiple regression analysis was conducted for all the variables (CD, gender, age, BMI, educational level, place of residence, and employment status) as determinants of FNS score and it revealed a significant effect (*p* = 0.0002). However, the only factor that was revealed as a significant determinant in this model was the presence of CD (*p* < 0.0001; β = 0.31), confirming previously indicated results for the single variable analysis.

## 4. Discussion

The findings of the present study indicate that the presence of CD is an important determinant of the FNS score, which was observed both in the general group of patients and in the major sub-groups. Despite the fact that the issue of food neophobia is relatively new and was not part of the routine assessment among the groups of patients diagnosed with diet-related diseases and who are recommended to follow a specific dietary recommendation, some studies were conducted in this area, but studies in relation to CD have not been carried out till date. It was indicated that in these patients, food neophobia may contribute to lower diet adherence and worse disease management, as was observed for individuals with type 1 diabetes [41]. However, as the present study involved comparison between two groups that followed a GFD, it must be emphasized that a risk associated with higher food neophobia exists in the CD-GFD group and may contribute to an unbalanced diet, although further studies including assessment of diet quality, variety of food products, and analysis of GFD adherence are needed. 

The fact that individuals with CD had a higher food neophobia level than other respondents following a GFD may be due to the fear of risk associated with the disease and the fact that they may be scared of adverse reactions to gluten-contaminated food products, which may be attributed to the risk of contamination and safety of products that are not certified as gluten-free [42]. The high level of food neophobia is associated with an increased correlation between choice and familiarity [43], therefore if CD patients are not familiar with the products, they may reject them. As a result, it may be indicated that CD patients may not have sufficient knowledge to make informed decisions with no fear of being exposed to gluten, and producers and restaurants do not facilitate such decisions [44]. In a study by Halmos et al. [45], which was conducted in a group of over 5000 CD patients, a problem with regard to poor nutritional knowledge was observed, and the patients also faced difficulties in recognizing products that are deemed to be safe for them, though in general they can describe sources of gluten. Furthermore, our previous study indicated that CD patients may be confused with the labeling of a number of food products [46], resulting in an unintentional consumption of gluten-containing products by the patients [47]. In addition, a GFD is commonly not balanced properly, and even if the patients adhere to the diet strictly, it may lead to serious consequences, such as poor nutritional status, cardiovascular problems, metabolic syndrome, and poor intestinal microbiota [48]. Such problems result in a serious emotional burden for CD patients [49] and may explain the observed higher food neophobia status among CD patients than for other respondents following a GFD based on their own decision. This is associated with the fact that following a GFD has now become a fashion [50], and a number of individuals follow it with no justified reason and no strict adherence [51], hence their food neophobia status is not influenced by the disease. 

In addition, findings from the present study indicate that despite the fact that individuals with CD are characterized by higher food neophobia levels (higher numeric value of FNS) than individuals following a GFD based on their own decision, the number of individuals in each FNS category is similar. It was observed that about 15% of respondents in each group were characterized by high FNS scores, when compared with the group-dependent median value. These findings are consistent with those reported by a Polish study, by Kozioł-Kozakowska et al. [52], conducted in a group of healthy pediatric individuals, which established that high food neophobia levels were observed in 12.3% of children.

While comparing the obtained results with those of Satherley et al. [25], which also assessed FNS scores of CD individuals, it must be emphasized that they did not calculate the proportion of individuals showing low, medium, and high FNS scores, owing to the fact that the aim of their study was to develop a CD Food Attitudes and Behaviours Scale (CD-FAB), to identify disordered eating attitudes and behaviors, but not presentation of the FNS scores of participants. Despite the fact, that CD-FAB is brief, self-report questionnaire that shows good reliability and validity in measuring disordered eating attitudes and behaviors in CD patients, it covers a lot of themes exploring food attitudes, concerns, and eating behaviors (i.e., handling of food, trust, risk-taking, and food safety), whereas the FNS is focused only on one narrow aspect. The CD-FAB could measure the relationship between attitudes, concerns, and eating behaviors and quality of life, but it was observed that total CD-FAB is positively correlated with the FNS [24]. The FNS is, at the same time, closely linked to adverse eating patterns and reduced dietary quality, which is related to an increased risk of obesity and non-communicable diseases which was proven in the recent study of Sarin et al. [24]. It must be emphasized, that in literature there are also other tools to measure traits related to the quality of life of CD patients and other aspects, such as the Celiac Dietary Adherence Test (CDAT) [53,54], Celiac Disease Self-Efficacy (Celiac-SE) questionnaire [55] or Celiac Disease Questionnaire (CDQ) [56]. In spite of the fact that the FNS and CD-FAB are different tools, it must be emphasized that common elements exist here, which are associated with identifying eating attitudes and behaviors, as it was demonstrated that food neophobic individuals have not only a tendency to avoid new food products, but also to dislike them [57] or to evaluate them in a negative way [18].

Considering the fact that the food neophobia levels may be categorized in various ways, mainly as mean ± SD [36,39,40] and also as specific cut-off points [57] or tertiles [58], the FNS scores should also be compared, if the data are available. In the present study, a mean ± SD value of 39.4 ± 9.2 was observed for the CD-GFD group, while the value was 33.6 ± 8.7 for the non-CD-GFD group. FNS scores for the Canadian population were found to be 29.6 ± 0.70 (mean ± SE) [59] and 34.5 ± 11.9 (mean ± SD) [28], for South Korean population it was 33.0 ± 10.1 (mean ± SD) [37], for the Belgian population it was 30.6 ± 9.4 (mean ± SD) [38], for the Finnish population they were 33.9 ± 11.4 (mean ± SD) [40] and 38.0 ± 10.5 (mean ± SD) [60], for the Dutch population it was 30.1 ± 9.5 (mean ± SD) [61], and for Australian rural students the value was 34.7 ± 0.64 (mean ± SE) and for urban students it was 29.35 ± 0.38 (mean ± SE) [58]. When comparing results of this study with those reported by other authors, obtained for healthy adults, it may be concluded that the results obtained for the CD-GFD group were definitely higher than expected for healthy individuals, indicating a higher food neophobia level, while results of the non-CD-GFD group were more typical, but also quite high.

Regardless of the differences in the FNS scores of adults from various countries, which may be influenced by demographic differences and country-specific differences [62], other factors influencing the FNS value should be indicated, which are also relevant for CD individuals and other individuals following a GFD. Some of the factors identified are familiarity with food products (important for following of a GFD) [40], beliefs (which may be associated with the gluten content) [63], sensory properties (different in the case of GFPs), [63] and disgust (which may be caused by the need to include products not consumed previously to the diet) [64]. All the indicated factors may cause higher food neophobia levels in CD patients and hence may also influence their adherence behavior.

Moreover, the factors associated with the course of the disease should also be considered as potential factors influencing food neophobia status. In a study by Olabi et al. [65], it was observed that negative food-related experiences while consuming a novel food product might increase an individual’s food neophobia level. This observation could be crucial for the individuals suffering from CD, as they have a risk of developing steatorrhea, diarrhea, and other gastrointestinal symptoms after the consumption of gluten (even if it is unintentional with contaminated food or unconscious) [66]. Therefore, negative food experiences associated with the intake of new and unknown food products which are gluten-contaminated may influence the food neophobia level in CD individuals. It should be emphasized that this finding is consistent with the finding of Satherley et al. [21], who stated that the FNS score in CD individuals is not a good predictor of disordered eating behavior, as food neophobia should be treated as some level of lack of trust, regardless of the reasons, which for CD individuals may be associated with the course of the disease and not with disordered eating habits. 

Food neophobia leads to the consumption of a limited variety of food products and compromised diet quality [59] and has also been previously linked to nutritional risk and increased risk of diet-related chronic diseases [40,67]. Therefore, it is important to introduce effective dietary strategies and impart education to CD individuals, not only to reduce the food neophobia levels but also to improve diet quality, including a variety of food products to provide a properly balanced diet, even if food neophobia is observed.

Except for the interesting novel observations of food neophobia in a group of CD patients, some limitations of the study must be indicated. Due to a strict inclusion criteria, as well as a relatively small number of diagnosed CD patients in Poland, the sample size was quite small. Moreover, while the CD-GFD group was quite homogeneous, it must be emphasized that, the non-CD-GFD group which followed a GFD based on their own decision was less homogeneous. Also, the lack of a control group of healthy subjects not following a GFD is a potential limitation to conclude about food neophobia of CD patients. Moreover, the survey was conducted via CD associations and GFD associations, therefore this approach should be indicated as a potential bias, due to the fact that it automatically excludes individuals not being members of CD or GFD associations. 

Other factors, specific for CD patients, that were not analyzed in the present study, such as time since diagnosis and severity of symptoms after gluten consumption, may also have influenced the obtained results, so further investigations are needed.

## 5. Conclusions

For the individuals following a GFD, CD is indicated as the major determinant contributing to higher food neophobia levels among CD individuals, compared to those following a GFD based on their own decision. The influence of other variables, such as gender, age, BMI, educational level, place of residence, and employment status was not observed in the assessed groups, neither for CD participants nor for those following a GFD based on their own decision. It may be concluded that CD is a major contributor to food neophobia, which can be attributed to fear of developing adverse reactions to gluten-contaminated food products, which is more pronounced in CD patients compared to non-CD patients following a GFD based on their own decision.

## Figures and Tables

**Table 1:** Characteristics of the study subjects.

Characteristics		Total (*n* = 225)	CD-GFD Group (*n* = 101)	Non-CD-GFD Group (*n* = 124)	*p*-Value
Gender	Male	26 (11.6%)	14 (13.9%)	12 (9.7%)	0.4432
	Female	199 (88.4%)	87 (86.1%)	112 (90.3%)	
Age (years)	Mean ± SD	36.4 ± 9.5	35.1 ± 10.2	37.5 ± 8.7	0.0132
	Median (range)	36.0 * (18.0–72.0)	35.0 * (19.0–72.0)	37.0 (18.0–60.0)	
BMI (kg/m^2^)	Mean ± SD	22.7 ± 3.8	22.4 ± 3.2	22.9 ± 4.2	0.4978
	Median (range)	22.0 * (16.5–42.4)	21.6 * (16.5–32.7)	22.3 * (17.1–42.4)	
BMI category	Underweight	15 (6.7)	7 (6.9)	8 (6.5)	0.9025
	Normal weight	169 (75.1)	77 (76.2)	92 (74.2)	
	Excessive body mass	41 (18.2)	17 (16.9)	24 (19.4)	
Educational level	Primary and secondary	44 (19.6)	23 (22.8)	21 (16.9)	0.5472
	Tertiary education	28 (12.4)	12 (11.9)	16 (12.9)	
	University degree	153 (68.0)	66 (65.3)	87 (70.2)	
Place of residence	Village	33 (14.7)	17 (16.8)	16 (12.9)	0.3992
	Town (<20,000 residents)	29 (12.9)	9 (8.9)	20 (16.1)	
	Cities (20,000–100,000 residents)	38 (16.9)	17 (16.8)	21 (16.9)	
	Cities (>100,000 residents)	125 (55.5)	58 (57.4)	67 (54.0)	
Employment status	Employed	173 (76.9)	77 (76.2)	96 (77.4)	0.9563
	Unemployed, students, and retired	52 (23.1)	24 (23.8)	28 (22.6)	

CD—eliac disease; GFD—gluten-free diet; BMI—body mass index; * non-parametric distribution.

**Table 2:** Food Neophobia Scale (FNS) scores of the respondents in sub-groups of CD-GFD and non-CD-GFD individuals.

FNS		Total	CD-GFD Group	Non-CD-GFD Group	*p*-Value
Score	Mean ± SD	36.2 ± 9.4	39.4 ± 9.2	33.6 ± 8.7	<0.0001
	95% CI	35.0–37.4	37.5–41.2	32.0–35.1	
	Median	36.0	40.0	34.0	
	Min–max	16.0–67.0	19.0–67.0	16.0–58.0	
	25th–75th	30.0–43.0	34.0–45.0	27.5–39.5	
Category	Low level	107 (47.6)	49 (48.5%)	58 (46.8%)	0.8869
	Average level	84 (37.3)	36 (35.6%)	48 (38.7%)	
	High level	34 (15.1)	16 (15.8%)	18 (14.5%)	

CD—celiac disease; GFD—gluten-free diet.

**Table 3:** Food Neophobia Scale (FNS) scores stratified by gender in the sub-groups of CD-GFD and non-CD-GFD individuals (mean ± SD and median accompanied by range).

Category	Total	CD-GFD Group	Non-CD-GFD Group	*p*-Value
Female	35.0 ± 9.3 36 * (16–67)	38.9 ± 9.6 39 (19–67)	34.1 ± 8.6 35 (22–45)	0.0002
Male	34.9 ± 9.3 36 (17–53)	42.2 ± 6.1 43 (29–53)	29.3 ± 8.6 29 (18–58)	0.0002
*p*-Value	0.8427	0.2185	0.0681	–

CD—celiac disease; GFD—gluten-free diet; * non-parametric distribution.

**Table 4:** The results of correlation analysis between Food Neophobia Scale (FNS) scores and age, as well as BMI, in the sub-groups of CD-GFD and non-CD-GFD individuals.

		Total	CD-GFD Group	Non-CD-GFD Group
Age	*p*	0.2241 *	0.5660 *	0.2160
	*R*	0.1233	0.0578	0.1118
BMI	*p*	0.8515 *	0.4272 *	0.6854 *
	*R*	−0.0114	−0.0719	0.0245

BMI—body mass index; * non-parametric distribution.

**Table 5:** Food Neophobia Scale (FNS) scores stratified by BMI in the sub-groups of CD-GFD and non-CD-GFD individuals (mean ± SD and median accompanied by range).

Category	Total	CD-GFD Group	Non-CD-GFD Group	*p*-Value
Underweight (BMI <18.5 kg/m^2^)	38.0 ± 10.9 38 (21–64)	40.1 ± 14 40 (21–64)	36.1 ± 7.7 38 (23–47)	0.4950
Normal weight (BMI 18.5–24.9 kg/m^2^)	36.1 ± 9.4 36 (16–67)	39.2 ± 9.1 39 (19–67)	33.5 ± 8.9 34 (16–55)	<0.0001
Excessive body mass (BMI >25 kg/m^2^)	35.9 ± 9.1 36 (18–58)	39.9 ± 8.8 43 (23–51)	33.0 ± 8.4 32.5 (18–58)	0.0138
*p*-Value	0.7298	0.9312	0.6691	–

CD—celiac disease; GFD—gluten-free diet; BMI—body mass index.

**Table 6:** Food Neophobia Scale (FNS) scores stratified by educational level in the sub-groups of CD-GFD and non-CD-GFD individuals (mean ± SD and median accompanied by range).

Category	Total	CD-GFD Group	Non-CD-GFD Group	*p*-Value
Primary and secondary	38.4 ± 10.0 37 (19–64)	40.8 ± 9.8 41 (22–64)	35.8 ± 9.7 36 (19–55)	0.0925
Tertiary education	33.9 ± 10.0 36 (16–53)	37.8 ± 11 40 (21–53)	31.0 ± 8.1 32 (16–43)	0.0697
University degree	36.0 ± 9.0 36 (17–67)	39.2 ± 8.8 39 (19–67)	33.5 ± 8.5 34 (17–58)	<0.0001
*p*-Value	0.1231	0.6306	0.2553	–

CD—celiac disease; GFD—gluten-free diet.

**Table 7:** Food Neophobia Scale (FNS) scores stratified by place of residence in the sub-groups of CD-GFD and non-CD-GFD individuals (mean ± SD and median accompanied by range).

Category	Total	CD-GFD Group	Non-CD-GFD Group	*p*-Value
Village	37.5 ± 8.6 36 (21–55)	40.1 ± 7.9 40 (23–53)	34.6 ± 8.5 35 (21–55)	0.0646
Town (<20,000 residents)	37.1 ± 7.9 37 (16–55)	40.7 ± 9.9 41 (24–55)	35.6 ± 6.4 37 * (16–43)	0.1791
Cities (20,000–100,000 residents)	36.6 ± 9.7 37 (19–67)	37.9 ± 11.1 39 (21–67)	35.5 ± 8.6 36 (19–52)	0.4531
Cities (>100,000 residents)	35.5 ± 9.8 36 (17–64)	39.4 ± 9.1 40 (19–64)	32.1 ± 9.2 32 (17–58)	<0.0001
*p*-Value	0.6558	0.8776	0.1439	–

CD—celiac disease; GFD—gluten-free diet; * non-parametric distribution.

**Table 8:** Food Neophobia Scale (FNS) scores stratified by employment status in the sub-groups of CD-GFD and non-CD-GFD individuals (mean ± SD and median accompanied by range).

Category	Total	CD-GFD Group	Non-CD-GFD Group	*p*-Value
Employed	36.2 ± 9.6 36 (17–67)	39.7 ± 9.2 40 (19–67)	33.4 ± 8.9 34 (17–58)	<0.0001
Unemployed, students, and retired	36.1 ± 8.8 37 (16–53)	38.5 ± 9.3 41 (21–53)	34.1 ± 8.0 36 (16–46)	0.0780
*p*-Value	0.9606	0.5774	0.7025	–

CD—celiac disease; GFD—gluten-free diet.
